# Hospice use and end‐of‐life care among older patients with esophageal cancer

**DOI:** 10.1002/hsr2.76

**Published:** 2018-07-19

**Authors:** Angela C. Tramontano, Ryan Nipp, Chung Yin Kong, Divya Yerramilli, Justin F. Gainor, Chin Hur

**Affiliations:** ^1^ Institute for Technology Assessment Massachusetts General Hospital Boston MA USA; ^2^ Department of Medicine, Division of Hematology and Oncology Massachusetts General Hospital Cancer Center & Harvard Medical School Boston MA USA; ^3^ Harvard Medical School Boston MA USA; ^4^ Department of Radiation Oncology Massachusetts General Hospital Boston MA USA

**Keywords:** end of life, esophageal cancer, hospice, outcomes, SEER‐Medicare

## Abstract

**Background:**

Hospice and end‐of‐life health care utilization among patients with esophageal cancer are understudied. We used the Surveillance, Epidemiology, and End Results (SEER)‐Medicare linked database to analyze hospice use and end‐of‐life treatment patterns.

**Methods:**

We included patients diagnosed with esophageal adenocarcinoma or squamous cell carcinoma between 2000 and 2011 and who had died by December 31, 2013. We evaluated patterns of hospice enrollment, chemotherapy receipt, radiation receipt, acute care hospitalizations, and intensive care unit (ICU) admissions at end of life. We used multivariate logistic regression to evaluate possible associations with hospice use, late ICU admission, and late chemotherapy receipt.

**Results:**

Our study included 6449 patients; 3597 (55.8%) enrolled in hospice. Among hospice enrolled patients, 31.4% enrolled in the last 7 days of life. Hospice enrollment increased over time, from 43.2% in 2000 to 59.6% in 2013. Patients who were older, female, with stage IV disease, or those with higher socioeconomic status were more likely to enroll in hospice. Among all patients, 19.1% had an ICU admission within the last 30 days and 4.6% received chemotherapy within the last 14 days of life. Those who were Black or Asian (compared to White), married, or had a comorbidity score >1 were more likely to have a late ICU admission. Males and younger patients were more likely to receive chemotherapy at end of life.

**Conclusion:**

Hospice enrollment rates among patients with esophageal cancer have increased over time; however, a significant percentage of patients enrolls near the end of life. Further research is needed to improve understanding of how end‐of‐life care decisions for these patients are made.

## 1 INTRODUCTION

Esophageal cancer incidence in the United States has risen over the past 2 decades, with an estimated 16 940 new cases and 15 690 deaths expected in 2017.^1^, ^2^ Despite recent advances in treatment options, the overall survival outlook for these patients remains poor, with a 5‐year survival of just 18.8%.^1^ While those with localized disease experience better 5‐year survival rates (42.9%), approximately 39% of patients are diagnosed with metastatic disease.^3^ Thus, as incidence continues to climb, efforts to improve health care delivery and outcomes for these patients are critically needed.

Previous studies have shown that hospice enrollment among patients with cancer has been increasing, but a substantial percentage still do not receive hospice services, or receive services near the end of life.^4^, ^5^, ^6^ Conversely, aggressive end‐of‐life cancer treatment has been steadily increasing, despite often providing limited benefit.^7^, ^8^ Notably, hospice care, sometimes considered under the purview of palliative care, is often defined as a service that provides comprehensive care for patients with terminal illness and their families, to provide services ranging from symptom management to bereavement.^9^ Importantly, hospice services can help manage the difficult symptoms patients may experience and provide greater psychological support.^10^, ^11^, ^12^ In addition, patients who have had end‐of‐life discussions regarding palliative and hospice care are more likely to use the services rather than undergo aggressive end‐of‐life treatment.^11^, ^13^ Therefore, despite improvements in hospice utilization and clear evidence supporting the benefits of hospice services, ongoing research is needed to identify ways of ensuring that patients receive appropriate referral for hospice services to alleviate suffering and improve end‐of‐life care.

Patients with esophageal cancer experience high rates of pain, dyspnea,^14^ and dysphagia^15^; however, the best management of these patients at the end of life remains unclear. Notably, little research exists to describe their patterns of hospice utilization. We sought to study patients with esophageal cancer to examine trends in hospice enrollment over time using the Surveillance, Epidemiology, and End Results (SEER)‐Medicare linked database. In addition, we aimed to determine which patient and clinical characteristics were associated with hospice enrollment and aggressive end‐of‐life treatment, defined as acute care hospitalizations within the last 30 days, intensive care unit (ICU) admissions within the last 30 days, radiation receipt within the last 14 days, and chemotherapy receipt within the last 14 days of life.^16^

## 2 METHODS

### 2.1 Inclusion and exclusion

We used the SEER‐Medicare linked database to estimate factors that predict hospice use and aggressive end‐of‐life treatment receipt among patients with esophageal cancer. The SEER database includes information about tumor characteristics for approximately 28% of the US population. The SEER‐Medicare database links SEER registry information with Medicare enrollment and claims. These files include inpatient, outpatient, and physician services, and are used to examine health care patterns over time.^17^

We included patients diagnosed at age 66 or older between January 1, 2000 and December 31, 2011, with esophageal adenocarcinoma (EAC) or esophageal squamous cell (ESC) as the primary cancer, and who had died by December 31, 2013. Histology was defined using International Classification of Diseases for Oncology (ICD‐O‐3) codes (Appendix A). Patients were included if they had continuous enrollment in Medicare Parts A and B from 13 months prior to their diagnosis to death. Patients were excluded if they had been enrolled in an HMO during this period to ensure that we captured complete claims data for health services. We based cancer stage on the SEER stage variable for the sixth edition of the AJCC Cancer Staging Manual. Those diagnosed prior to 2004 were mapped using the SEER variables for extension of disease and lymph node involvement to the appropriate AJCC sixth edition stage; patients with unknown stages were excluded.

### 2.2 Statistical analysis

We examined patient and clinical characteristics that may predict use of hospice and end‐of‐life treatment: age, sex, race/ethnicity (White, Black, Hispanic, Asian/Other), marital status, SEER region (Northeast, South, Midwest, West/Hawaii), urban location (big metropolitan, metropolitan/urban, less urban/rural), ecological socioeconomic (SES) status, AJCC stages (I, II, III, IV), and comorbidity score (0, 1, 2+). We imputed ecological SES status using ZIP code‐level median household income from US census data provided in SEER‐Medicare to derive quintiles. Charlson comorbidity scores were calculated using the Deyo adaptation of the Charlson comorbidity index for the 13‐month period prior to cancer diagnosis.^18^, ^19^, ^20^ Survival was defined as the time from diagnosis date to date of death.

We classified patients as having an ICU admission if they had an ICU claim on the inpatient Medicare file. Patients were classified as having received chemotherapy, radiation, or surgery if they had at least 1 claim based on codes in Appendix A. Aggressive end‐of‐life treatment was defined using indicators previously established as measures of end‐of‐life care by the National Quality Forum, as well as earlier work on indicators of end‐of‐life care in claims data: an ICU admission within the last 30 days, any acute care hospitalizations within the last 30 days, at least 2 acute care hospitalizations within the last 30 days, chemotherapy receipt within the last 14 days, or radiation receipt within the last 14 days of life.^16^ Patients were defined as enrolled in hospice if they had at least 1 hospice claim between diagnosis and death in the Medicare hospice claims file. Patients were defined as having late hospice enrollment if their first hospice claim occurred within 7 days of death.

We used chi‐square tests to compare the distribution of patient characteristics among patients with and without hospice enrollment. We examined the hospice enrollment and end‐of‐life treatment prevalence by year and used Cochran‐Armitage tests to analyze trends over time. Multivariable logistic regression models were used to identify associations between patient and clinical characteristics and hospice enrollment in the entire cohort and late hospice enrollment among hospice enrolled patients. We used multivariate logistic regression to analyze 2 indicators of aggressive treatment: chemotherapy receipt within the last 14 days of life and ICU admission within the last 30 days of life, for a total of 4 separate multivariate models. To correct for false positive determinations of significance that can occur when running multiple tests, we applied a Bonferroni correction for each of the 4 models. We used a *P* value of 0.0125 (0.05/4) to test the significance of the model; if significant, a *P* value <.05 was used to determine significance of the independent variables.

Statistical significance was defined as *P* value <.05 in a 2‐sided test. Analyses were performed using SAS software, version 9.4 (SAS Institute, Inc., Cary, NC).

### 2.3 Ethical considerations

This study has been approved as exempt by the Institutional Review Board at Massachusetts General Hospital. A Data Use Agreement was signed before obtaining the data from SEER‐Medicare.

## 3 RESULTS

### 3.1 Characteristics

The cohort included 6449 patients: 3994 (61.9%) with EAC and 2455 (38.1%) with ESC (Table 1). The majority were male (4740; 73.5%) and white race (5538; 85.9%); 2201 (34.1%) patients had stage IV disease. Over half were married (3658; 56.7%), or lived in a large metropolitan area (3359; 52.1%). We found that 1620 (25.1%) received surgery, 4118 (63.9%) received radiation, 3423 (53.1%) received chemotherapy, and 3683 (57.1%) had an ICU admission at any time after diagnosis. The median (interquartile range [IQR]) age at death was 77 (72‐82). The median (IQR) survival was 14.7 months (3.5‐17.3) for the entire cohort.

**Table 1:** Characteristics of patients diagnosed with esophageal cancer between 2000 and 2011

Characteristic	Entire Cohort (N = 6449)	Any Hospice (N = 3597)	No Hospice (N = 2852)
Age (at death)			
66‐69	847 (13.1%)	454 (12.6%)	393 (13.8%)
70‐74	1617 (25.1%)	878 (24.4%)	739 (25.9%)
75‐79	1701 (26.5%)	931 (25.9%)	776 (27.2%)
80‐84	1256 (19.5%)	723 (20.1%)	533 (18.7%)
85+	1022 (15.9%)	611 (17%)	411 (14.4%)
Sex			
Male	4740 (73.5%)	2593 (72.1%)	2147 (75.3%)
Female	1709 (26.5%)	1004 (27.9%)	705 (24.7%)
Race/ethnicity			
White	5538 (85.9%)	3154 (87.7%)	2384 (83.6%)
Black	569 (8.8%)	282 (7.8%)	287 (10.1%)
Hispanic	92 (1.4%)	49 (1.4%)	43 (1.5%)
Asian/other^a^	250 (3.9%)	112 (3.1%)	138 (4.8%)
Marital status			
Unmarried	2791 (34.3%)	1401 (39.0%)	1166 (40.9%)
Married	3658 (56.7%)	2068 (57.5%)	1590 (55.8%)
Unknown	224 (3.5%)	128 (3.6%)	96 (3.4%)
AJCC stage			
I	1339 (20.8%)	695 (19.3%)	644 (22.6%)
II	1386 (21.5%)	699 (19.4%)	687 (24.1%)
III	1523 (23.6%)	819 (22.8%)	704 (24.7%)
IV	2201 (34.1%)	1384 (38.5%)	817 (28.7%)
Charlson score			
0	2843 (44.1%)	1628 (45.3%)	1215 (42.6%)
1	1849 (28.7%)	1053 (29.3%)	796 (27.9%)
2+	1757 (27.2%)	916 (25.5%)	841 (29.5%)
Year of death			
2000‐2004	2032 (31.5%)	1016 (28.3%)	1016 (35.6%)
2005‐2008	2289 (35.5%)	1298 (36.1%)	991 (34.8%)
2009‐2013	2128 (33%)	1283 (35.7%)	845 (29.6%)
SEER region			
Northeast	1423 (22.1%)	730 (20.3%)	693 (24.3%)
South	1567 (24.3%)	928 (25.8%)	639 (22.4%)
Midwest	880 (13.7%)	561 (15.6%)	319 (11.2%)
West/Hawaii	2579 (40%)	1378 (38.3%)	1201 (42.1%)
Place of residence			
Large metropolitan	3359 (52.1%)	1825 (50.7%)	1534 (53.8%)
Metro/urban	2497 (38.7%)	1432 (39.8%)	1065 (37.3%)
Less urban/rural	593 (9.2%)	340 (9.5%)	253 (8.9%)
SES (census tract quintile)			
0 (lowest)	1245 (19.3%)	645 (17.9%)	600 (21.0%)
1	1273 (19.7%)	676 (18.8%)	597 (20.9%)
2	1283 (19.9%)	690 (19.2%)	593 (20.8%)
3	1289 (20%)	761 (21.2%)	528 (18.5%)
4 (highest)	1359 (21.1%)	825 (22.9%)	534 (18.7%)
Histology			
Adenocarcinoma	3994 (61.9%)	2299 (63.9%)	1695 (59.4%)
Squamous	2455 (38.1%)	1298 (36.1%)	1157 (40.6%)
Surgery			
No	4829 (74.9%)	2812 (78.2%)	2017 (70.7%)
Yes	1620 (25.1%)	785 (21.8%)	835 (29.3%)
Radiation			
No	2331 (36.2%)	1314 (36.5%)	1017 (35.7%)
Yes	4118 (63.9%)	2283 (63.5%)	1835 (64.3%)
Chemotherapy			
No	3026 (46.9%)	1677 (46.6%)	1349 (47.3%)
Yes	3423 (53.1%)	1920 (53.4%)	1503 (52.7%)
ICU			
No	2766 (42.9%)	1761 (49%)	1005 (35.2%)
Yes	3683 (57.1%)	1836 (51%)	1847 (64.8%)

a Includes 28 patients of a race/ethnicity other than Asian.

### 3.2 Hospice enrollment

**Figure 1:**
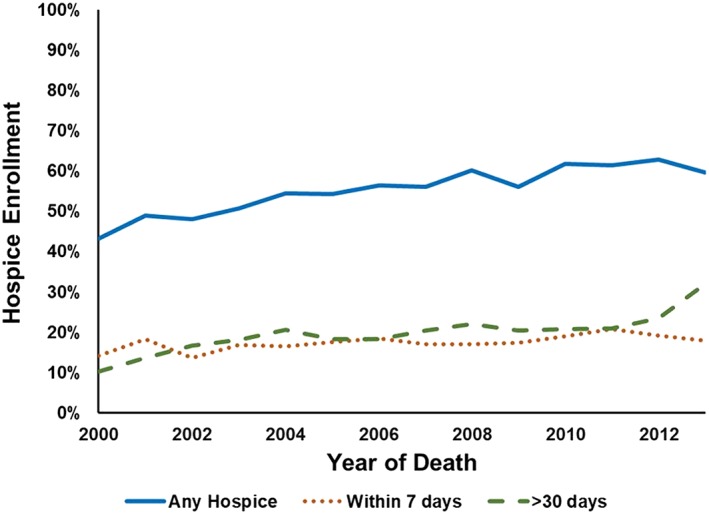
Hospice enrollment: All, <7, and >30 days before death

Over half of patients enrolled in hospice (3597; 55.8%). The percentage of patients who enrolled in hospice showed a steady increase over time from 43.2% in 2000 to 59.6% in 2013 (Cochran‐Armitage test for trend, *P* < .0001) (Figure 1). Among enrolled hospice patients, 31.4% did not enroll until 7 days before death, demonstrating a high rate of late enrollment. Notably, 6.1% of patients who enrolled in hospice lived more than 180 days on hospice. The median (IQR) age at death was similar for the 2 groups, with 77 (72‐82) for those enrolled in hospice and 76 (82‐72) for those who were never enrolled. Patients who were enrolled in hospice had a longer median survival than those who never enrolled (8.8 months (IQR 3.9‐17.9) vs 6.9 months (2.9‐16.4); Wilcoxon‐Mann‐Whitney *P* < .0001).

Our multivariable logistic regression model found that patients were more likely to have enrolled in hospice if they were 80 years or older, female, had stage IV disease at the time of diagnosis, died in later years of the study period, lived in either the South or Midwest, or had a SES quintile >2 (Table 2; Wald chi‐square *P* < .05). Additionally, Asian patients were less likely to have enrolled in hospice compared to White patients (odds ratio [OR]: 0.69; 95% CI 0.53‐0.90; Wald chi‐square *P* = .006), and those with a Charlson score >1 were less likely to have enrolled than those with a score = 0 (OR: 0.82; 95% CI 0.72‐0.93; Wald chi‐square *P* = .002). We ran a multivariable logistic regression model to determine factors among those who enrolled in hospice that may predict late enrollment, defined as within 7 days of death. Our model showed that patients who were 75 years old or older, female, lived in the South or West/Hawaii (compared to Northeast), or lived in a metro/urban area (compared to larger metro) were less likely to have a late enrollment.

**Table 2:** Characteristics associated with hospice enrollment among esophageal cancer patients, 2000 to 2013^a^

Characteristic	Hospice Enrollment (N = 3597)^b^		Late Hospice Enrollment (N = 1131)^b^	
	OR (95% CI)	*P* Value^c^	OR (95% CI)	*P* Value^c^
Age (at death)				
66‐69	1.00		1.00	
70‐74	1.09 (0.92‐1.29)	.33	0.86 (0.68‐1.10)	.23
75‐79	1.12 (0.94‐1.32)	.21	0.77 (0.60‐0.98)	.04
80‐84	1.28 (1.07‐1.54)	.008	0.67 (0.52‐0.87)	.003
85+	1.47 (1.21‐1.80)	.0001	0.69 (0.52‐0.91)	.008
Sex				
Male	1.00		1.00	
Female	1.28 (1.13‐1.45)	.0001	0.76 (0.63‐0.90)	.002
Race/ethnicity				
White	1.00		1.00	
Black	0.83 (0.68‐1.02)	.07	0.89 (0.65‐1.22)	.47
Hispanic	1.02 (0.67‐1.56)	.93	1.20 (0.65‐2.21)	.55
Asian/other	0.69 (0.53‐0.90)	.006	1.04 (0.69‐1.60)	.85
Marital status				
Unmarried	1.00		1.00	
Married	1.08 (0.97‐1.21)	.16	1.14 (0.98‐1.34)	.10
Unknown	1.11 (0.84‐1.48)	.44	1.05 (0.70‐1.58)	.8
AJCC stage				
I	1.00		1.00	
II	0.95 (0.81‐1.10)	.48	0.95 (0.75‐1.21)	.69
III	1.15 (0.99‐1.34)	.08	0.99 (0.78‐1.24)	.90
IV	1.69 (1.45‐1.96)	<.0001	0.996 (0.80‐1.24)	.97
Charlson score				
0	1.00		1.00	
1	1.00 (0.89‐1.13)	.98	0.86 (0.73‐1.02)	.09
2+	0.82 (0.72‐0.93)	.002	1.03 (0.86‐1.23)	.76
Year of death				
2000‐2004	1.00		1.00	
2005‐2008	1.30 (1.15‐1.47)	<.0001	0.95 (0.79‐1.14)	.56
2009‐2013	1.51 (1.32‐1.72)	<.0001	1.01 (0.83‐1.21)	.96
SEER region				
Northeast	1.00		1.00	
South	1.48 (1.26‐1.73)	<.0001	0.57 (0.46‐0.72)	<.0001
Midwest	1.68 (1.40‐2.01)	<.0001	0.83 (0.65‐1.05)	.11
West/Hawaii	1.08 (0.94‐1.23)	.28	0.66 (0.54‐0.80)	<.0001
Place of residence				
Large metropolitan	1.00		1.00	
Metro/urban	1.12 (1.00‐1.24)	.0499	0.82 (0.70‐0.96)	.01
Less urban/rural	1.02 (0.84‐1.24)	.88	0.77 (0.58‐1.02)	.07
SES (census tract quintile)				
0 (lowest)	1.00		1.00	
1	0.99 (0.84‐1.17)	.91	1.26 (0.99‐1.60)	.06
2	0.98 (0.84‐1.16)	.85	1.13 (0.89‐1.45)	.32
3	1.22 (1.03‐1.44)	.02	0.97 (0.76‐1.24)	.80
4 (highest)	1.28 (1.08‐1.52)	.004	1.15 (0.90‐1.47)	.26
Histology				
Adenocarcinoma	1.00		1.00	
Squamous	0.92 (0.82‐1.03)	.14	0.96 (0.81‐1.13)	.60
Survival (months)	1.000 (0.997‐1.003)	.89	0.996 (0.99‐1.001)	.13

a Model significant after Bonferroni correction.

b c‐statistic: 0.62 (hospice), 0.60 (late hospice).

c Wald chi‐square.

Patients who die soon after diagnosis may not have an opportunity to enroll. To determine whether there were differences in enrollment rates among those with a shortened survival, we compared those who died within 30 days of diagnosis (285; 4.4%) to those who lived longer (6164; 95.6%). We found those who died within 30 days were less likely to enroll in hospice compared to those who lived more than 30 days after diagnosis (48.1% vs 56.1%; chi‐square *P* = .007).

We also looked at rates of hospice use among patients who did not receive any treatment (surgery, radiation, and/or chemotherapy) to those who did. There were 1273 (19.7%) who received no treatment; they were more likely to have enrolled in hospice (61.0% vs 54.5%; *P* < .0001) and were also more likely to have metastatic disease (45.6% vs 31.3%%; *P* < .0001).

### 3.3 Aggressive end‐of‐life treatment

**Figure 2:**
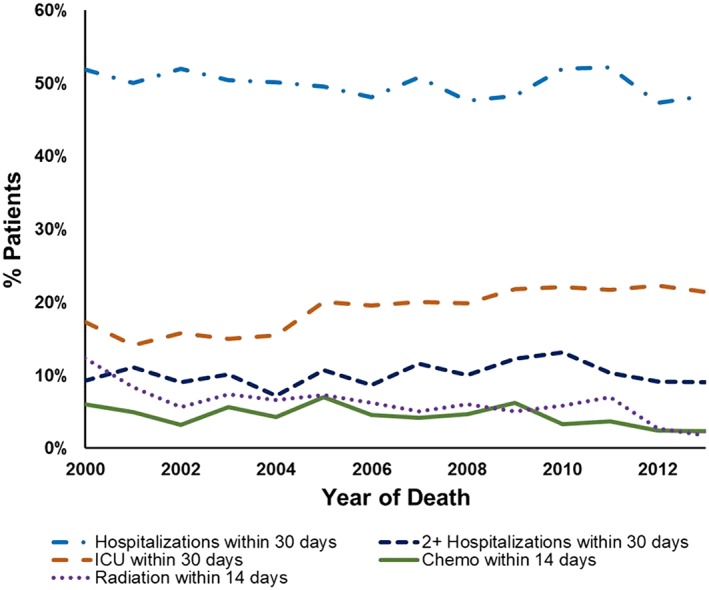
Late treatment, 2000 to 2013 period

We used 5 distinct modalities as separate indicators of aggressive end‐of‐life care: ICU admissions, any hospitalization, or at least 2 hospitalizations within 30 days of death, and chemotherapy or radiation receipt within 14 days of death. Overall, 1231 (19.1%) had a late ICU admission, 3218 (49.9%) had a late acute care hospitalization, 661 (10.2%) had at least 2 late acute care hospitalizations, 295 (4.6%) received late chemotherapy, and 400 (4.2%) received late radiation. Figure 2 shows rates over time. The percentage of patients who had any acute care hospitalization remained steady over time (51.9% in 2000 to 48.3% in 2013; Cochran‐Armitage test for trend, *P* = .55); the percentage of patients with 2 or more hospital admissions also remained steady over time (9.2% in 2000 to 9.0% in 2013; Cochran‐Armitage test for trend, *P* = .23). However, the percentage of patients admitted to the ICU increased from 17.3% in 2000 to 21.4% in 2013 (Cochran‐Armitage test for trend, *P* < .0001). In contrast, the percentage of patients receiving late chemotherapy decreased over time from 6.0% in 2000 to 2.3% in 2013 (Cochran‐Armitage test for trend, *P* = .03); late radiation receipt also decreased over time from 12.4% in 2000 to 1.7% in 2013 (Cochran‐Armitage test for trend, *P* < .0001). Those who had an ICU admission within 30 days of death were also more likely to receive late chemotherapy (6.9% vs 4.0%; chi‐square *P* < .0001). For both aggressive treatment indicators, those who received the treatment were less likely to be enrolled in hospice than those who did not (27.2% vs 62.5%; chi‐square *P* < .0001 for late ICU; 21.4% vs 57.4%; chi‐square *P* < .0001 for late chemotherapy).

We used 2 separate multivariable logistic regression models to further analyze 2 indicators of aggressive end‐of‐life care (late ICU admissions and late chemotherapy receipt) and determine associations between patient and clinical characteristics and these modalities (Table 3). In our ICU admission adjusted model, patients who were Black or Asian, age 66 to 69 (compared to 85+), married, had a comorbidity score >0, lived in a large metropolitan area, or died in later years were more likely to be admitted to the ICU within 30 days of death. Those with ESC or stage IV disease (vs stage I) were less likely to be admitted to the ICU. In our adjusted model of late chemotherapy treatment, patients who were 80 years or older, female, or had shorter survival were less likely to receive chemotherapy within 14 days of death (Wald chi‐square *P* < .003). There were no other statistically significant associations in the late chemotherapy model.

**Table 3:** Characteristics associated with end‐of‐life aggressive treatment among esophageal cancer patients, 2000 to 2013^a^

	ICU Admission Within Last 30 days (N = 1231)^b^		Chemotherapy Within Last 14 days (N = 295)^b^	
Characteristic	OR (95% CI)^a^	*P* Value^c^	OR (95% CI)	*P* Value^c^
Age (at death)				
66‐69	1.00		1.00	
70‐74	0.87 (0.71‐1.08)	.21	0.86 (0.61‐1.22)	.39
75‐79	0.86 (0.69‐1.06)	.15	1.01 (0.72‐1.42)	.96
80‐84	0.80 (0.64‐1.002)	.052	0.49 (0.31‐0.76)	.001
85+	0.58 (0.45‐0.74)	<.0001	0.38 (0.22‐0.65)	.0004
Sex				
Male	1.00		1.00	
Female	0.97 (0.83‐1.13)	.68	0.57 (0.41‐0.80)	.001
Race/ethnicity				
White	1.00		1.00	
Black	1.40 (2.20‐2.79)	.007	0.63 (0.38‐1.07)	.09
Hispanic	1.48 (0.91‐2.42)	.12	0.23 (0.03‐1.65)	.14
Asian/other	1.57 (1.25‐2.37)	.004	0.57 (0.27‐1.19)	.13
Marital status				
Unmarried	1.00		1.00	
Married	1.20 (1.04‐1.38)	.01	1.09 (0.84‐1.42)	.51
Unknown	0.98 (0.67‐1.41)	.91	0.43 (0.15‐1.19)	.10
AJCC stage				
I	1.00		1.00	
II	1.02 (0.84‐1.23)	.86	0.92 (0.60‐1.42)	.72
III	0.90 (0.74‐1.09)	.26	1.17 (0.79‐1.73)	.44
IV	0.59 (0.48‐0.71)	<.0001	1.35 (0.93‐1.95)	.11
Charlson score				
0	1.00		1.00	
1	1.15 (0.99‐1.35)	.07	1.02 (0.77‐1.35)	.88
2+	1.34 (1.15‐1.57)	.0002	0.87 (0.64‐1.18)	.36
Year of death				
2000‐2004	1.00		1.00	
2005‐2008	1.40 (1.19‐1.65)	<.0001	1.19 (0.90‐1.58)	.22
2009‐2013	1.66 (1.40‐1.97)	<.0001	0.97 (0.71‐1.33)	.85
SEER region				
Northeast	1.00		1.00	
South	0.81 (0.66‐0.99)	.04	1.03 (0.71‐1.50)	.88
Midwest	0.82 (0.65‐1.02)	.08	1.12 (0.71‐1.70)	.59
West/Hawaii	0.96 (0.81‐1.13)	.60	1.15 (0.83‐1.59)	.40
Place of residence				
Large metropolitan	1.00		1.00	
Metro/urban	0.62 (0.54‐0.72)	<.0001	0.98 (0.76‐1.26)	.87
Less urban/rural	0.54 (0.41‐0.71)	<.0001	0.83 (0.52‐1.35)	.46
SES (census tract quintile)				
0 (lowest)	1.00		1.00	
1	1.08 (0.88‐1.32)	.48	1.02 (0.89‐1.52)	.94
2	1.04 (0.84‐1.28)	.72	1.15 (0.78‐1.72)	.48
3	0.83 (0.67‐1.03)	.09	1.18 (0.79‐1.76)	.41
4 (highest)	0.85 (0.69‐1.05)	.14	1.07 (0.71‐1.61)	.74
Histology				
Adenocarcinoma	1.00		1.00	
Squamous	0.85 (0.73‐0.99)	.03	1.19 (0.91‐1.55)	.21
Survival (months)	0.998 (0.99‐1.001)	.17	0.99 (0.98‐0.997)	.01

a Model significant after Bonferroni correction.

b c‐statistic: 0.63 (ICU), 0.67 (chemotherapy).

c Wald chi‐square.

## 4 DISCUSSION

We analyzed trends in hospice utilization and aggressive treatment at end of life among older patients with esophageal cancer using the linked SEER‐Medicare database. We found that while most (55.8%) patients enrolled in hospice, a significant percentage of enrollees received hospice services quite close to their date of death (31.4% within 7 days of death). In addition, nearly half of patients never enrolled in hospice. Notably, studies suggest that early integration of palliative care may enhance hospice enrollment rates and also diminish rates of late hospice enrollment among patients with cancer, and, thus, our findings further highlight the need for efforts to integrate palliative care services earlier in the disease course for patients with advanced esophageal cancer.^11^, ^21^, ^22^ Importantly, hospice use increased over time from 2000 to 2013, which is consistent with studies of hospice enrollment trends among patients with other types of cancer,^5^, ^6^, ^23^, ^24^ and also aligns with recommendations from guidelines and expert groups.^25^, ^26^, ^27^ The overall rate of hospice utilization among patients with esophageal cancer was slightly lower than those found in overall Medicare cancer patients (59.5% in 2009).^6^ It is also lower than rates seen in other cancer sites during a similar period, such as hepatocellular carcinoma (63.0%),^28^ breast cancer (62.8%),^29^ and glioma (63.0%)^30^ but higher than others, such as leukemia (44.4%).^24^ Collectively, our data support the need for additional research to understand barriers to hospice enrollment and to help encourage appropriate use of hospice services for patients with esophageal cancer.

Our analysis found that nearly one‐half of patients with esophageal cancer were admitted to an acute care hospital within 30 days of death and 10% of our cohort had at least 2 hospital admissions. These rates remained steady over time, demonstrating that aggressive end‐of‐life care continues to remain high in this population. One‐fifth of patients had an ICU admission within 30 days of death, and nearly 5% received chemotherapy within 14 days of death. Female patients were more likely to enroll in hospice and less likely to receive aggressive end‐of‐life treatment, which may suggest differences in how patients approach treatment decisions. Black patients were more likely to receive aggressive treatment than White patients, and Asian patients were less likely to enroll in hospice. Notably, these sex^7^, ^31^ and racial/ethnic differences are consistent with other studies on disparities in end‐of‐life care^31^, ^32^, and these findings further corroborate the need for efforts to address disparities in end‐of‐life care for patients with cancer. Interestingly, we found that marital status was not associated with hospice enrollment or late chemotherapy use, similar to an earlier study among patients with cancer.^32^

Earlier studies have demonstrated that hospice enrollment differs based on geographic location.^33^ Our study also suggests regional and socioeconomic differences in hospice enrollment and end‐of‐life treatment. Hospice enrollment was higher among patients within a higher ecological SES quintile, and among patients who lived in the South or Midwest (compared to Northeast). This contrasts with a similar study that showed no association between income and hospice enrollment in an adjusted model.^32^ Patients living in the South or Midwest or living outside a large metropolitan area were less likely to have a late ICU admission. The mechanism for these findings requires additional study, but our results highlight that regional and socioeconomic differences play a role in patents' end‐of‐life care.

Patients diagnosed with stage IV cancer were more likely to enroll in hospice and less likely to have a late ICU admission. Clinically, this aligns with the fact that patients with esophageal cancer who are diagnosed with metastatic disease may have fewer treatment options than those diagnosed with earlier stages. It is possible that these patients receive information about hospice as a health care option earlier in their disease trajectory and at higher rates than earlier stage patients. Alternatively, some earlier stage patients who ultimately experience disease progression may experience a more rapid decline, thus representing a barrier to early discussions about hospice enrollment. Interestingly, we found that patients who enrolled in hospice had a longer median survival than those who never enrolled, and this is hypothesis‐generating, but likely related to the fact that those who survived a longer period also had more time to be enrolled on hospice.

Our study has several limitations. It only includes patients older than 65 who resided in a SEER region and received Medicare services. Therefore, our results may not be generalizable to other populations. However, most patients with esophageal cancer are diagnosed above age 65 (60%), and SEER data represents one of the largest population databases available.^1^ We identified patients who had died and analyzed health care utilization before death, which may be subject to biases.^34^ For example, older cancer patients may die from treatment complications, and it is not possible to determine whether physicians knew a patient's terminal status. However, this retrospective method allows us to readily identify all patients who died and analyze their end‐of‐life care. Our analysis of health care use is based on Medicare claims, but the treatments may not accurately reflect patient preferences or physician recommendations. In addition, those patients who choose hospice enrollment may be more likely to forego more aggressive treatment options. This study reflects treatment patterns prior to the publication of the CROSS trial (2012) and may not be generalizable to locally advanced patients because of changes in management^36^; it also does not include newer immunotherapy treatment modalities.^37^ Furthermore, we are unable to determine whether the end‐of‐life care was given as a palliative treatment or with curative intent.

Our study has multiple strengths. To our knowledge, it is the first population‐based study to focus on end‐of‐life heath care utilization among patients with esophageal cancer. In addition, this database allowed us to analyze treatment and hospice enrollment trends over time. Importantly, use of the large SEER‐Medicare database allowed us to adjust for many factors that may influence end‐of‐life treatment and hospice use, such as sociodemographics and comorbidities.

In summary, our study demonstrates that hospice enrollment among older patients with esophageal cancer has been steadily increasing over time. However, a substantial proportion of patients continue to receive no hospice services, and many enroll very shortly prior to their death. Patients with esophageal cancer may experience a substantial symptom burden due to their treatment or disease progression, and this can be distressing for patients and their family. Thus, earlier initiation of palliative care and/or appropriate hospice services has the potential to enhance care delivery and outcomes for these patients and their loved ones. Notably, we found that while late chemotherapy rates may be decreasing over time ICU admission rates continue to increase. Collectively, our findings suggest that there may be more room for improvement, and further research is needed to determine how to provide the best possible care at the end of life for patients with esophageal cancer.

## FUNDING

NIH grant R01 CA140574.

## CONFLICT OF INTEREST

JFG served as a compensated consultant or received honoraria from Bristol‐Myers Squibb, Novartis, Genentech/Roche, Merck, Incyte, Pfizer, Loxo, Theravance, Array Biopharm, and Ariad/Takeda. There are no other conflicts of interest.

## AUTHOR CONTRIBUTIONS

Conceptualization: ACT, RN, CH

Formal analysis: ACT, RN, CH, CYK, DY, JFG

Writing—original draft preparation: ACT, RN, CH

Writing—review and editing: ACT, RN, CH, CYK, DY, JFG

## APPENDIX A Table A1: SEER‐medicare claims codes

**Table:**

Variable	Source	Codes
Adenocarcinoma diagnosis	ICD‐0‐3 codes	8050, 8140‐8147, 8160‐8162, 8180‐8221, 8250‐8507, 8514, 8520‐8551, 8560, 8570‐8574, 8576, 8940‐8941
Squamous diagnosis	ICD‐0‐3 codes	8070‐8078, 8083, 8084
Surgery	CPT or ICD‐9 procedure codes	43100, 43101, 43107, 43108, 43112, 43113, 43116, 43117, 43118, 43119, 43121, 43122, 43123, 43124, 43217, 43216, 43228, 43250, 43251, 43257, 43258, 96570, 96571, 42.33
Radiation	CPT or ICD‐9 procedure codes	ICD‐9‐CM: V58.0, 92.21‐92.29 CPT: 7331‐7336, 73399, 77400‐77499, 77750‐77799 Revenue center: 0330, 0333, 0339
Chemotherapy	CPT or ICD‐9 procedure codes	ICD‐9‐CM: V58.1, 99.25 HCPCS: C1166, C1168, C1179, C9110, C9205, C9207, C9213‐C9216, C9411, C9414‐C9419, C942x, C9430‐C9438, G0345‐G0363, J9000‐J9999, Q0083‐Q0085 CPT: 9651x‐9654x, 964xx Revenue center 0331, 0332, 0335
